# A new family with an activating mutation (G431S) in the TSH receptor gene: a phenotype discussion and review of the literature

**DOI:** 10.1186/1687-9856-2014-23

**Published:** 2014-11-17

**Authors:** Cæcilie C Larsen, Lefkothea P Karaviti, Victor Seghers, Roy E Weiss, Samuel Refetoff, Alexandra M Dumitrescu

**Affiliations:** Department of Medicine, The University of Chicago, Chicago, IL 60637 USA; Department of Pediatric, Texas Children’s Hospital, Houston, TX 77030 USA; Department of Radiology, Texas Children’s Hospital, Houston, TX 77030 USA; Department of Medicine, University of Miami, Miami, FL 33101 USA; Department of Pediatrics, The University of Chicago, Chicago, IL 60637 USA; Committee on Genetics, The University of Chicago, Chicago, IL 60637 USA

**Keywords:** Hyperthyroidism, Nonautoimmune, Pediatric, Radioiodine, Thyrotropin, Receptor mutation, Activating

## Abstract

Germline nonautoimmune hyperthyroidism due to an activating mutation in the *thyroid stimulating hormone receptor* gene is an uncommon disease. To date 32 different mutations have been described. The severity of the hyperthyroid symptoms is variable and phenotype differences have been described in subjects harboring the same mutation. This paper describes a family with a mutation in codon 431 of the *thyroid stimulating hormone receptor* gene. This is the most common activating mutation in the *thyroid stimulating hormone receptor* gene with total of 13 patients harboring the mutation in four families. The similarities and differences among patients with the mutation in codon 431 are discussed. Furthermore all previously reported activating mutations in the *thyroid stimulating hormone receptor* gene are reviewed.

**Electronic supplementary material:**

The online version of this article (doi:10.1186/1687-9856-2014-23) contains supplementary material, which is available to authorized users.

## Background

The thyroid stimulating hormone receptor (TSHR) belongs to the family of rhodopsin-like G protein-coupled receptor (GPCR). It consists of a large extracellular domain with a binding site for thyroid stimulating hormone (TSH), seven transmembrane domains and a small intracytoplasmic tail [1]. The TSHR has a pivotal role in regulating the growth of thyrocytes and the synthesis of thyroxine (T_4_) and triiodothyronine (T_3_) by stimulating mainly the cAMP pathway.

The first case of familial non-autoimmune hyperthyroidism (NAH) was published in 1982 [2]. In 1994 the mechanism of NAH was identified by Duprez et al., who described the first activating mutation in the *TSHR* gene [3]. The disease is relatively uncommon and, so far, 32 different mutations and 147 patients have been reported. Most of the mutations are located in the transmembrane domains encoded by exons 9 and 10 of the *TSHR* gene. Reported cases of gain of function *TSHR* gene mutations are summarized in Additional file 1: Table S1 (familial) and Additional file 2: Table S2 (sporadic).

Patients with NAH present with classic symptoms of hyperthyroidism, including weight loss, goiter, hyperactive behavior and tachycardia. The severity of the hyperthyroid symptoms is variable. This has differed among patients from different families and even among affected individuals belonging to the same family and harboring the same mutation [4]. Some patients present with mental retardation and craniosynostosis whereas other patients were reported to only have subclinical hyperthyroidism [5, 6]. Many could not be controlled on antithyroid drugs and needed surgery and/or radioactive iodine treatment [7].

Herein, we describe a family from North America in which an activating mutant TSHR (Gly431Ser) was identified in the propositus, his older brother and mother, all suffering from NAH. The two boys were treated with radioactive iodine and to our knowledge the propositus is the youngest patient with NAH that received this modality of treatment.

## Case presentation

**Figure 1:**
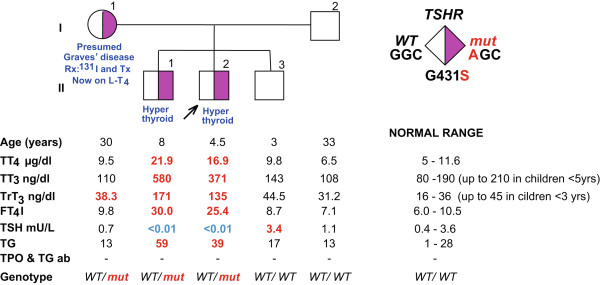
**Pedigree and TFTs.** Square symbols indicate males, circles females. Results of thyroid function test are aligned with each symbol and were obtained on no hormonal replacement. Roman numerals to the left of the pedigree indicate the generation and numerals on the right of each symbol indicate individual family member. Abnormal values in bold, high in red and low in blue. Rx, treatment; Tx, thyroidectomy; ^131^I, radioactive iodine.

The propositus II-2 (Figure 1), a 4.5 years old boy, came to the attention of one of us (LPK) because of signs of hyperthyroidism and a family history of thyroid disease, suspicious for NAH. At 3 years of age he presented with low body weight, failure to thrive and had difficulties gaining weight even though he had a good appetite, prompting an initial referral to gastroenterology. He was born at term to a mother of white European background (English and German). Birth weight was 2721 g and neonatal thyroid screening was reported as normal. Physical exam revealed tachycardia, tremor and a hyperdynamic precordium. He was also noticed to be hyperactive but without any signs of developmental problems. Records from birth to 24 months show a head circumference at the 25th percentile. He had prominently appearing eyes but no true exophthalmos. Ultrasound showed a mild enlargement of the thyroid gland, the right lobe being 4.0 × 1.5 × 0.9 cm and the left lobe 3.9 × 1.2 × 0.7 cm., with hypoechoic foci consistent with small nodules. Thyroid function tests (TFTs), demonstrated thyroid hormone excess without detectable thyroid stimulating immunoglobulin (TSI) and he was initially placed on methimazole. In addition, he had elevated 24-hour radioiodide uptake of 67% (normal range 15-25%), confirming the endogenous source of the thyroid hormone. Bone age was within 2SD of the chronological age. Blood samples from the patient and members of the family were sent to the Thyroid Study Unit at the University of Chicago for further evaluation. TFTs measured in our laboratory showed high total T_4_ (TT_4_) of 16.9 μg/dl (reference range 5–11.6) and a total T_3_ (TT_3_) of 371 ng/dl (reference range for age 95–210), together with a suppressed TSH and negative thyroperoxidase (TPO) and thyroglobulin (TG) antibodies (see Figure 1), suspicious for NAH.

An older brother II-1 (Figure 1) was found to have the same clinical picture as the propositus. It is not clear when he began to have symptoms of hyperthyroidism but according to the mother, he had always been thin while growing up. His birth weight and length were in the normal range. The TFTs results obtained were also compatible with hyperthyroidism with negative TSI and anti-TPO/TG antibodies. Ultrasound showed a mild enlargement of the thyroid gland, the right lobe being 5.2 × 1.4 × 2.3 cm and the left lobe, 4.7 × 1.3 × 1.7 cm, with hypoechoic foci consistent with small nodules. 24-hour radioiodide uptake was high at 90% (normal range 15-25%). Bone age was advanced to 12 years at a chronological age of 7 years, being 4SD above the chronological age. He also had hyperactive behaviour but no signs of mental retardation. Records from birth to 24 months show a head circumference at the 50th percentile.

Family history revealed that the mother (I-1, Figure 1) underwent radioactive iodide treatment at age 18 because of symptoms of hyperthyroidism and presumed Graves’ disease. She was 16–17 years old when she noted tachycardia, tremor and restlessness, and she states to have been always thin. Following radioiodide ablation she was placed on thyroid hormone replacement. After her third delivery she had a thyroidectomy because of the presence of thyroid nodules in the regenerated thyroid gland. We were not able to get information about additional family members on the maternal side of the family. The father (I-2), and a younger brother (II-3) did not have any signs of hyperthyroidism and had normal TFTs (see Figure 1).

After the diagnosis of NAH was confirmed, the two boys underwent treatment with radioactive iodine to ablate the overactive thyroid gland. The propositus and older brother received doses of 12.7 mCi and 14.1 mCi of I-131, respectively. Both children became hypothyroid and were started on L-T_4_ treatment. One year after the I-131 treatment, on 25 and 50 μg of L-T_4_ daily, respectively, the propositus and his older brother had TT_3_ of 100 and 88 (90-260 ng.dL), TT_4_ of 9.7 and 9 (4.5-12 μg/dl) and TSH of 0.43 and 2.64 mU/L (0.5-4.3 mIU/L), respectively. The L-T4 doses were further titrated subsequently. The hyperactive behavior in the two brothers disappeared, their school performance improved and both have been gaining weight.

## Materials and methods

Blood was collected locally and shipped at room temperature for detailed analyses at the Thyroid Study Unit at the University of Chicago. TT_4_, TT_3_, and TSH were measured by chemiluminescence immunometric assays using the Elecsys Automated System (Roche Molecular Biochemicals GmbH and Hitachi, Ltd., Indianapolis, IN). Total reverse T_3_ (TrT_3_) was measured by RIA (Adaltis Italia S.p.A, Bologna, Italy), and TG by an in-house RIA. Serum free T_3_ index (FT_3_I) and FT_4_I were calculated as the product of the total serum concentrations of each iodothyronine and the normalized resin T_4_ uptake ratio. Antibodies to TG and TPO were measured by passive hemagglutination (Fujirebio, Inc., Tokyo, Japan).

Genomic DNA was extracted from whole blood using the QIA amp DNA blood mini kit (Qiagen). Exons 1–10 in the *TSHR* were amplified by the polymerase chain reaction (PCR) with 14 sets of primers described earlier [8].

The conditions for the PCR were an initial ‘touchdown’ with denaturation for 30 s (94°C), annealing for 45 s (63°C) and elongation for 45 s (72°C) for 7 cycles, followed by denaturation for 30 s (94°C), annealing for 30 s (57.5°C) and elongation for 45 s (72°C) for 30 cycles and a final elongation for 7 minutes (72 °C).

All PCR products were electrophoresed on a 1.8% agarose gels and ϕX174/Hae III fragments (New England Biolabs) were used as molecular weight marker. Sequencing was carried out by automated fluorescence-based sequencer (373OXL 96 capillary, Applied Biosystem Carlsbad, CA).

## Results and discussion

The pedigree and TFTs of the propositus and his family members are shown in Figure 1. Results of the propositus, his brother and mother were compatible with familial NAH. Sequencing of the *TSHR* gene revealed a single nucleotide substitution in codon 431, where a normal guanosine is replaced by an adenosine resulting in the replacement of the normal glycine with a serine, (G431S). This mutation was first described in 2001 by Biebermann et al. [9]. Their in vitro studies demonstrated that expression of the mutant TSHR in COS-7 cells resulted in a greater cAMP accumulation compared to that in cells expressing the WT TSHR. The same was true for the basal inositol trisphosphate formation. The propositus’ father (I-2) and the youngest brother (II-3) did not harbor the mutation and had normal TFTs.

The hyperthyroidism of the propositus (II-2) and his older brother (II-1) could not be controlled with antithyroid drugs. This is a common finding among patients with NAH. In fact, 47/144 patients with *TSHR* gene mutations (Additional file 1: Tables S1 and Additional file 2: Table S2) causing gain-of-function received either thyroidectomy or radioactive iodine ablation. They were eventually both treated with radioactive iodide, at the age of almost five years and eight years, respectively. Currently they are both hypothyroid on L-T_4_ treatment.

There are big geographic differences when it comes to the use of radioactive iodide as a treatment option for children with hyperthyroidism and there have been concerns about the long-term risk of malignancies. However, in a study by Read et al. [10], of 107 children (<20 years) who received radioiodide, 98 had a follow-up period of 36 years and none developed thyroid cancer or leukemia. Further, it is known that the other alternative, namely thyroidectomy, is associated with a higher complication rate in children than in adults, especially if the surgery is not performed by a specialized and experienced thyroid surgeon [11]. In addition to having higher complication rates in children, surgery is not curative as the remaining thyroid cells continue to be stimulated by the activating TSHR mutation and hyperthyroidism often recurs. Then repeated surgeries or ablation with radioactive iodine are needed for definitive treatment.

The guidelines from the American Thyroid Association for children with Graves disease are to consider a definitive treatment with surgery or I-131 ablation if remission is not induced after 1–2 years on anti-thyroid drugs [12]. No specific guidelines are available for the treatment of children with hyperthyroidism caused by germline gain of function mutations in the *TSHR* gene. The management is on a case-by-case basis and depends on the severity of the mutations in terms of the degree of hyperthyroidism. Anti-thyroid drugs do not induce remission in these cases, and lifelong treatment with these drugs would be required. Therefore, a more definitive treatment is needed early on in NAH. When the goiter is large and higher I-131 doses would be required for ablation, surgery can be considered first. Often, this is followed by repeated surgeries or I-131treatment when hyperthyroidism recurs from the residual thyroid cells being stimulated by the *TSHR* gene mutation (Additional file 1: Tables S1 and Additional file 2: Table S2). In absence of large goiters, ablation with radioactive iodine is considered a definitive treatment, as it was the case for the two boys reported herein.

The propositus and his older brother were noted to have hyperactive behavior, which improved after treatment. They were not mentally retarded even though this is a feared complication of NAH. Among the previously reported patients, mental retardation was diagnosed in 5.6% of the patients (8/144, Additional file 1: Tables S1 and Additional file 2: Table S2). Among those with mental retardation, the age of diagnosis was no later than 24 months emphasizing the importance of early diagnosis of hyperthyroidism caused by NAH. Importantly all but one of these patients went on to receive a definitive treatment to prevent further complications. Bieberman et al. has also reported a patient with NAH who had a hyperactive behavior that improved with treatment [13].

In rare instances, germline gain of function *TSHR* gene mutations can cause severe hyperthyroidism (Additional file 1: Table S1). Depending on the mutation this can be severe from the beginning or it can worsen over time, as the gland continues to grow and overproduce thyroid hormones due to stimulation by the activating mutant TSHR. Features of fetal hyperthyroidism include intrauterine growth retardation, fetal hydrops, and craniosynostosis [14]. Features of hyperthyroidism in neonates include hyperkinesis, diarrhea, poor weight gain, vomiting, and in severe cases cardiac failure, arrhythmias, hypertension, hepatosplenomegaly and craniosynostosis [14, 15]. Manifestations of hyperthyroidism in childhood include poor weight gain, advanced bone age, hyperactive behavior, tachycardia [15]. Psychomotor disabilities can be present if the hyperthyroidism has caused craniosynostosis with microcephaly [15]. Of note, the more advanced bone age in the older brother of the propositus is due to the longer duration of the hyperthyroidism.

Both the propositus and his older brother were noted to have staring eyes. In 1994, Duprez et al. excluded from the characteristics of NAH the presence of autoimmune signs such as exophthalmos. However, several of the patients have been reported with eye symptoms (20/144). The pathogenesis of eye manifestations in NAH is not clear. The presence of the TSHR in the orbital tissue of Graves’ patients has been reported, thus the possibility that the mutant TSHR is located in orbital tissue [16]. Another possible explanation is the effect of thyroid hormones on the sympathetic nervous system, which controls the contraction of the ciliary muscle.

Including this family, four families with a total of 13 affected individuals, have been shown to harbor the G431S mutation (Table 1). This is the most common *TSHR* gene mutation in NAH occurring in unrelated families and not involving a CpG dinucleotide (consecutive cytosine and guanine bases separated by phosphate) mutational hot spot. In the four families, the age of diagnosis ranges from 3 to 18 years. These families have been reported between 2001 and current report. Their FT_4_ levels range from 133% to 286% expressed as percent of the upper limit of normal range. This indicates that environmental factors could be important for the phenotype expression. One patient was reported with premature birth but this was not the case with the propositus and his brother. In two out of the four families, eye manifestations, such as proptosis, were present, but of note, these families also had higher thyroid hormone levels. Except for one, all the patients needed thyroidectomy or radioiodine treatment. None had developmental abnormalities.

**Table 1:** **Clinical characteristics of the four families harboring the TSHR G431S mutation**

	***Number in pedigree***	***Reference***	***T***_***4***_ ***and T***_***3***_ ***(% upper limit)***	***Preterm birth (<37 wks)***	***Developmental problems (IQ test, speech delay)***	***Prominent eyes***	***Treatment (Age)***	***Age of diagnosis***	***F/M***
**Family 1**	I-1	[9]	N.a.	N.a.	N.a.	N.a.	Tx (15 y)	Youth	F
**Family 1**	II-1		N.a.	N.a.	N.a.	Yes	PTU, Tx (7 y)	4 y	M
**Family 1**	III-1 (proband)		FT4 = 275% FT3 = 293%	Yes, 36	N.a.	Yes, mildly	PTU, Tx (7 y)	3 y	M
**Family 2**	II-2	[17]	N.a.	N.a.	N.a.	N.a.	Tx	When IV-1 was diagnosed	N.a.
**Family 2**	III-1		N.a.	N.a.	N.a.	N.a.	Tx (18 y)	17 y	M
**Family 2**	III-3		N.a.	N.a.	N.a.	N.a.	Tx (15 y)	13 y	F
**Family 2**	IV-1		FT4 > 128% FT3 = 156%	No	N.a.	N.a.	MMI (7.5 y) Tx (8.4 y)	5 y	M
**Family 3**	B1	[6]	FT4 = 133% FT3 = 217%	N.a.	N.a.	N.a.	MMI, RAI (12 y)	5 y	M
**Family 3**	B2		FT4 = 204% FT3 = 199%	N.a.	N.a.	N.a.	MMI, RAI (15 y)	7 y	F
**Family 3**	Father		N.a.	N.a.	N.a.	N.a.	MMI	Hyperthyroidism in more than 15 years	M
**Family 4**	(I-1)		N.a.	N.a.	No	No	RAI (18 y), Tx (after 3. child)	16 y	F
**Family 4**	II-1		TT4 = 189% TT3 = 305%	No	Hyperactive behaviour which diminished after treatment	Yes	MMI, RAI (8 y)	8	M
**Family 4**	II-2 (propositus)		TT4 = 146% TT3 = 195%	No	Hyperactive behaviour which diminished after treatment	Yes	MMI, RAI (4.5 y)	4.5 y	M

Abbreviations: *N.a.* not available, *RAI* radioactive Iodine, *Tx* thyroidectomy, *F* female, *M* make, *PTU* propylthiouracil.

*MMI* Methimazole, *Y* year, *FT4* free T4, *FT3* free T3, *TT4* total T4 and *TT3* total T3.

Of note, the propositus’ mother was misdiagnosed as having Graves’ disease. Even though the clinical picture of Graves’ disease can be similar to that of NAH the distinction is important since the choice of treatment often differs and genetic counseling is important in NAH.

## Conclusion

In conclusion, we report a family with non-autoimmune hyperthyroidism due to an activating mutation in the *TSHR* gene, G431S. The affected members of this family were treated with radioactive iodine.

This case and the literature review on gain of function TSHR mutations illustrate that:

1. Lack of recognition of this rare disease has potential deleterious side effects in terms of brain development and metabolic issues that could lead to failure to thrive.
2. Careful consideration is required before initiating treatment with radioactive iodide at an early age considering the long-term risks. For other causes of hyperthyroidism in children surgery can be considered, especially for large goiters and when there is access to a high-volume thyroid surgeon. However, in the treatment of hyperthyroidism caused by gain of function TSHR mutations, surgery is not curative, and radioactive iodide is considered a definitive treatment.
3. Long-term follow-up is required as there is likelihood of relapse.
4. Sequencing analysis of the *TSHR* gene is required to confirm the diagnosis of NAH when clinically suspected.
5. A clinical lesson: the history of the mother’s hyperthyroidism and the index of suspicion triggered by her two children with failure to thrive prompted the evaluation of the proband in gastroenterology and then endocrinology.
6. It is important that the endocrine and pediatric communities are aware of this rare genetic condition.
7. In summary, we want to emphasize the need for early diagnosis and treatment of patients with NAH to avoid severe forms of hyperthyroidism with developmental abnormalities and craniosynostosis.

## Consent

Studies were approved by University of Chicago Institutional Review Board and all investigated family members gave their informed written consent.

## Electronic supplementary material

Additional file 1: Table S1: Summary of all reported patients with familial gain of function mutations in the TSHR. (DOCX 168 KB)

Additional file 2: Table S2: Summary of all reported patients with sporadic gain of function mutations in the TSHR. (DOCX 111 KB)

## Abbreviations

NAH: Nonautoimmune hyperthyroidism
RAI: Radioactive iodide
TFT: Thyroid function tests
TSH: Thyroid stimulating hormone
TSHR: Thyroid stimulating hormone receptor
Tx: Thyroidectomy.
