# Supplementary material for: A new family with an activating mutation (G431S) in the TSH receptor gene: a phenotype discussion and review of the literature
Source: Int J Pediatr Endocrinol. 2014 Nov 17;2014(1):23. doi: 10.1186/1687-9856-2014-23 (PMC4396564; doi:10.1186/1687-9856-2014-23)
Supplement: Supplementary file 1 — Additional file 1: Table S1: Summary of all reported patients with familial gain of function mutations in the TSHR. (DOCX 168 KB) [file 13633_2014_366_MOESM1_ESM.docx]

# Additional file 1: Table S1

| **Summary of all reported patients with familial gain of function mutations in the TSHR** | | | | | | | | | | |
| --- | --- | --- | --- | --- | --- | --- | --- | --- | --- | --- |
| ***Mutation*** | ***Number in pedi-gree*** | ***Refe-rence*** | ***Free T_4_ and free T_3_ (% upper limit)*** | ***Preterm birth (<37wks)*** | ***Developmental problems (IQ test, speech delay)*** | ***Prominent eyes*** | ***Treatment***  ***(Age)*** | ***Age of diagnosis*** | ***Craniosynosto-sis***  ***Head circumference (cm)*** | ***F/M*** |
| **Asn406Ser** | I-1 | (1) | T4 normal  T3 normal | N.a. | N.a. | N.a. | No | 53 y | N.a. | M |
| **Asn406Ser** | II-1 |  | T4=153%  T3=147% | N.a. | N.a. | N.a. | Potassium Iodide, RAI (28y) | 26 y | N.a. | M |
| **Gly431Ser** | I-1 | (2) | N.a. | N.a. | N.a. | N.a. | Tx (15y) | Youth | N.a. | F |
| **Gly431Ser** | II-1 |  | N.a. | N.a. | N.a. | Yes | PTU, Tx (7y) | 4 y | N.a. | M |
| **Gly431Ser** | III-1 |  | T4=275%  T3= 293% | Yes, 36 | N.a. | Yes, mildly | PTU, Tx (7y) | 3y | N.a. | M |
| **Gly431Ser** | II-2 | (3) | N.a. | N.a. | N.a. | N.a. | Tx | When IV-1 was diagnosed | N.a. | N.a. |
| **Gly431Ser** | III-1 |  | N.a. | N.a. | N.a. | N.a. | Tx (18y) | 17y | N.a. | M |
| **Gly431Ser** | III-3 |  | N.a. | N.a. | N.a. | N.a. | Tx (15y) | 13y | N.a. | F |
| **Gly431Ser** | IV-1 |  | T4>128%  T3=156% | No | N.a. | N.a. | MMI (7.5y)  Tx (8.4y) | 5y | N.a. | M |
| **Gly431Ser** | B1 | (4) | T4=133%  T3=217% | N.a. | N.a. | N.a. | MMI, RAI (12y) | 5y | N.a. | M |
| **Gly431Ser** | B2 |  | T4=204%  T3=199% | N.a. | N.a. | N.a. | MMI, RAI (15y) | 7y | N.a. | F |
| **Gly431Ser** | Father |  | N.a. | N.a. | N.a. | N.a. | MMI | Hyperthyroi-dism in more than 15 years | N.a. | M |
| **Met453Thr** | II-2 | (5) | N.a | N.a. | Normal | Yes | PTU, Tx (8 and 18y), RAI (18, 21 and 28y) | 0.1y | N.a | M |
| **Met453Thr** | III-2 |  | T4 normal  T3= 325% | Yes, 30 | Yes | Yes | PTU, Tx (4y) | 8 mo. | Normal | F |
| **Met453Thr** | III-3 |  | T4=153%  T3=108% | Yes, 34 | Normal | Yes | PTU | At births | Yes | M |
| **Met463Val** | IV-2 | (6) | N.a. | N.a. | N.a. | N.a. | Tx | 20y | N.a. | F |
| **Met463Val** | V-1 |  | N.a. | N.a. | N.a. | N.a. | Tx, MMI | 9y | N.a. | F |
| **Met463Val** | V-2 |  | N.a. | N.a. | N.a. | N.a. | Tx | 21y | N.a. | F |
| **Met463Val** | V-5 |  | N.a. | N.a. | N.a. | N.a. | MMI | 13y | N.a. | M |
| **Met463Val** | VI-1 |  | N.a. | N.a. | N.a. | N.a. | MMI | 7y | N.a. | M |
| **Met463Val** | VI-2 |  | T4=135%  T3=180% | N.a. | N.a. | N.a. | MMI | 5y | N.a. | M |
| **Met463Val** | VI-3 |  | T4 normal  T3= 144% | N.a. | N.a. | N.a. | MMI | 2y | N.a. | M |
| **Met463Val** | VI-4 |  | T4=268%  T3=220% | No | Normal | No | MMI | 4y | N.a. | F |
| **Met463Val** | I-2 | (7) | N.a. | N.a. | N.a. | N.a. | MMI | 30y | N.a. | F |
| **Met463Val** | II-2 |  | N.a. | N.a. | N.a. | N.a. | Tx (40y) | 27y | N.a. | M |
| **Met463Val** | II-3 |  | N.a. | N.a. | N.a. | N.a. | MMI, Tx (35y) | 18y | N.a. | M |
| **Met463Val** | III-1 |  | N.a. | N.a. | Normal | Yes mildly | MMI | 8y | N.a. | F |
| **Ala485Val** | I-1 | (8) | T4=717%  T3=111% | N.a. | Normal | No | Tx | 36 | N.a. | M |
| **Ala485Val** | II-1 |  | T4= 300%  T3=285% | No | Normal | No | PTU | 3.5 | N.a. | M |
| **Ala485Val** | II-2 |  | T4= 182%  T3=185% | No | Normal | No | PTU | Birth | N.a. | F |
| **Ser505Arg** | I-1 | (9) | T4>100% | N.a. | N.a. | N.a. | N.a. | N.a. |  | M |
| **Ser505Arg** | II-3 |  | N.a. | N.a. | N.a. | No | ATD, Tx | ? Youth | N.a. | F |
| **Ser505Arg** | III-2 |  | N.a. | N.a. | N.a. | No | ATD, Tx | 10y | N.a. | F |
| **Ser505Arg** | IV-1 |  | N.a. | N.a. | N.a. | No | ATD, Tx | 1y | N.a. | F |
| **Ser505Arg** | II-4 |  | N.a. | N.a. | N.a. | No | ATD, Tx | ? Youth | N.a. | M |
| **Ser505Arg** | II-6 |  | N.a. | N.a. | N.a. | No | ATD | ? Youth | N.a. | F |
| **Ser505Arg** | I-1 | (10) | T4=107% | N.a. | N.a. | N.a. | Tx (9y) | 35y | N.a. | M |
| **Ser505Arg** | II-2 |  | T4 normal | No | Normal | N.a. | No | 2mo | N.a. | F |
| **Ser505Asn** | II-1 | (11) | N.a. | N.a. | N.a. | Yes | MMI, Tx (13, 19), RAI (21y), MMI | 9y | N.a. | M |
| **Ser505Asn** | III-1 |  | TT4=153%  T3= 209% | Yes, 30 | N.a. | N.a. | MMI, Tx (8y) | 4y | 24cm (3^rd^ centile) | M |
| **Ser505Asn** | III-2 |  | TT4= 155%  T3=234% | Yes, 33 | Yes, motor and speech delay | No | MMI, Tx (6y), MMI | 18mo | 28cm (5^th^ centile) | F |
| **Val509Ala** | II-6 | (12) | T4>100% | N.a. | N.a. | No | ATD | 30y | N.a. | F |
| **Val509Ala** | II-7 |  | T4>100% | N.a. | N.a. | No | ATD | 34y | N.a. | F |
| **Val509Ala** | III-15 |  | T4>100% | N.a. | N.a. | No | N.a. | N.a. | N.a. | F |
| **Val509Ala** | III-18 |  | T4>100% | N.a. | N.a. | No | ATD | 21y | N.a. | F |
| **Val509Ala** | III-20 |  | T4>100% | N.a. | N.a. | No | ATD | 14y | N.a. | F |
| **Val509Ala** | III-22 |  | T4>100% | N.a. | N.a. | No | ATD, Tx | 13y | N.a. | M |
| **Val509Ala** | I-1 | (13) | N.a. | N.a. | N.a. | N.a. | Tx (60y) | N.a. | N.a. | F |
| **Val509Ala** | II-1 |  | N.a. | N.a. | N.a. | N.a. | Tx (36y) | 18y, but symptoms since childhood | N.a. | M |
| **Val509Ala** | III-1 |  | T4=163%  T3=109% | N.a. | N.a. | N.a. | MMI | 4y | N.a. | M |
| **Ile568Val** | I-1 | (14) | N.a. | N.a. | N.a. | N.a. | Tx (25y) | 25y | N.a. | F |
| **Ile568Val** | II-1 |  | N.a. | N.a. | N.a. | N.a. | Tx (34y), RAI (38y) | 18y | N.a. | M |
| **Ile568Val** | III-1 |  | T4= 147%  T3=194% | N.a. | N.a. | N.a. | MMI | 16 y | N.a. | F |
| **Glu575Lys** | I-1 | (15) | T4=75%  T3=92% | N.a. | N.a. | N.a. | N.a. | 64 y | N.a. | F |
| **Glu575Lys** | II-1 |  | T4 normal  T3 normal | N.a. | N.a. | N.a. | N.a. | Adulthood | N.a. | M |
| **Glu575Lys** | II-2 |  | T4 normal  T3 normal | N.a. | N.a. | N.a. | N.a. | Adulthood | N.a. | M |
| **Val597Phe** | II-2 | (16) | N.a. | N.a. | N.a. | N.a. | Tx (18y) | 18 y | N.a. | M |
| **Val597Phe** | III-1 |  | N.a. | N.a. | N.a. | N.a. | Tx (9y) | 7 y | N.a. | F |
| **Val597Phe** | III-2 |  | T4=253%  T3=198% | N.a. | N.a. | No | MMI | 5 y | N.a. | F |
| **Asp617Tyr** | II-1 | (17) | T4 normal  T3 normal | N.a. | N.a. | N.a. | N.a. | 55 y | N.a. | F |
| **Asp617Tyr** | II-2 |  | T4=214 %  T3=198% | N.a. | N.a. | N.a. | Tx (48y) | 48 y | N.a. | F |
| **Asp617Tyr** | III-1 |  | T4=171%  T3=168& | N.a. | N.a. | N.a. | Potassium iodine | 21y | N.a. | F |
| **Asp617Tyr** | III-2 |  | T4 normal | N.a. | N.a. | N.a. | N.a. | 20y | N.a. | M |
| **Asp617Tyr** | III-3 |  | T4 normal | N.a. | N.a. | N.a. | N.a. | 24y | N.a. | F |
| **Asp617Tyr** | III-4 |  | T4=151%  T3=145% | N.a. | N.a. | N.a. | Potassium iodine | 20 y | N.a. | F |
| **Ala623Val** | III-9 | (18) | T4=176%  T3=201% | N.a | N.a | N.a | Tx twice, PTU | 3y | N.a. | F |
| **Ala623Val** | IV-11 |  | T4=212%  T3=201% | No | N.a. | Fixed eyes deviation | MMI | 3.5 mo. | 33 (10^th^) | M |
| **Ala623Val** | IV-12 |  | T4=188%  T3=193% | No | N.a. | N.a | MMI | 3.5 wks. | 31(3th) | M |
| **Met626Ile** | I-1 | (19) | FT4I=117%  TT3 normal | N.a. | N.a. | N.a. | Tx | N.a. | N.a. | F |
| **Met626Ile** | II-4 |  | FT4I=266%  TT3=316% | N.a. | N.a. | N.a. | N.a. | 30 y | N.a. | M |
| **Met626Ile** | III-2 |  | FT4I=110%  TT3=182% | No | N.a. | No | MMI | 10 mo. | N.a. | F |
| **Met626Ile** | III-3 |  | FT4I=190%  TT3=158% | Yes, 34wks | N.a. | No | N.a. | 15 mo. | N.a. | M |
| **Met626Ile** | III-4 |  | FT4I=200%  TT3=123% | N.a. | N.a. | N.a. | N.a. | Birth | N.a. | F |
| **Met626Ile** | III-1 | (20) | N.a. | N.a. | Normal | No | PTU, MMI, RAI (14y) | 10y | Normal | F |
| **Met626Ile** | IV-1 |  | T4=110% | No | Normal | No | PTU | 6wks | N.a. | M |
| **Leu629Phe** | II-4 | (21) | N.a. | N.a. | N.a. | N.a. | MMI (12y), Tx (17y), MMI (21y), RAI (25y) | 12 y | N.a. | F |
| **Leu629Phe** | III-1 |  | N.a.  TT3=360% | Yes, 33 | No | Yes | MMI | 2 y | Yes | M |
| **Phe631Ser** | I-1 | (22) | TT4=165%  TT3= 203% | N.a. | N.a. | Yes | MMI, RAI | 42 y | N.a. | F |
| **Phe631Ser** | II-1 |  | TT4=174% | Yes, 34 | N.a. | N.a. | MMI | 19 y | N.a. | M |
| **Phe631Ser** | II-2 |  | T4=324%  TT3= 278% | Yes, 34 | N.a. | N.a. | MMI | 11 y | N.a. | F |
| **Phe631Ser** | II-3 |  | T4=141%  TT3=196% | Yes, 30 | Cerebral palsy | N.a. | MMI | 10 y | N.a. | F |
| **Thr632Ile** | I-1 | (23) | N.a. | N.a. | N.a. | N.a. | N.a. | 12 y | N.a. | F |
| **Thr632Ile** | II-1 |  | N.a. | N.a. | N.a. | N.a. | N.a. | 7 mo. | Yes | N.a. |
| **Cys636Trp** | I-1 | (4) | N.a. | N.a. | N.a. | N.a. | MMI | N.a. | N.a. | F |
| **Cys636Trp** | II-1 |  | T4= 102%  T3=122% | N.a. | N.a. | N.a. | MMI | 5.5 y | N.a. | F |
| **Pro639Ser** | I-1 | (24) | N.a. | N.a. | N.a. | N.a. | MMI, Tx (41y) | 38y | N.a. | M |
| **Pro639Se** | II-1 |  | TT4=133% | No | Normal | Yes | MMI, Tx (21y) | 7 y | No | M |
| **Pro639Ser** | II-2 |  | N.a. | N.a. | Normal | Yes | MMI, Tx (20y), MMI | 5,5 y | N.a. | F |
| **Pro639Ser** | II-3 |  | TT4=114% | N.a. | N.a. | Yes | MMI, Tx (20y) | 5 y | N.a. | M |
| **Asn650Tyr** | I-1 | (25) | T4>100% | N.a. | N.a. | No | Tx | N.a. | N.a. | F |
| **Asn650Tyr** | II-2 |  | T4>100% | N.a. | N.a. | No | Tx | N.a. | N.a. | F |
| **Asn650Tyr** | II-3 |  | T4>100% | N.a. | N.a. | No | Tx | N.a. | N.a. | M |
| **Asn650Tyr** | III-1 |  | T4>100% | N.a. | N.a. | No | N.a. | 14y | N.a. | M |
| **Asn650Tyr** | III-2 |  | T4>100% | N.a. | N.a. | No | N.a. | 23y | N.a. | M |
| **Asn670Ser** | I-1 | (25) | T4>100% | N.a. | N.a. | No | N.a. | N.a. | N.a. | M |
| **Asn670Ser** | II-4 |  | T4>100% | N.a. | N.a. | No | N.a. | N.a. | N.a. | F |
| **Asn670Ser** | II-5 |  | T4>100% | N.a. | N.a. | No | N.a. | N.a. | N.a. | F |
| **Asn670Ser** | III-1 |  | T4>100% | N.a. | N.a. | No | ATD, RAI | 17y | N.a. | F |
| **Asn670Ser** | III-3 |  | T4>100% | N.a. | N.a. | No | N.a. | N.a. | N.a. | F |
| **Cys672Tyr** | I-1 | (12) | T4>100% | N.a. | N.a. | No | N.a. | N.a. | N.a. | F |
| **Cys672Tyr** | II-1 |  | T4>100% | N.a. | N.a. | No | N.a. | N.a. | N.a. | F |
| **Cys672Tyr** | II-2 |  | T4>100% | N.a. | N.a. | No | N.a. | N.a. | N.a. | M |
| **Cys672Tyr** | II-4 |  | T4>100% | N.a. | N.a. | No | N.a. | N.a. | N.a. | F |
| **Cys672Tyr** | II-5 |  | T4>100% | N.a. | N.a. | No | N.a. | N.a. | N.a. | F |
| **Cys672Tyr** | II-6 |  | T4>100% | N.a. | N.a. | No | Tx | 53y | N.a. | M |
| **Cys672Tyr** | III-1 |  | T4>100% | N.a. | N.a. | No | RAI | N.a. | N.a. | M |
| **Cys672Tyr** | III-3 |  | T4>100% | N.a. | N.a. | No | N.a. | N.a. | N.a. | M |
| **Cys672Tyr** | III-10 |  | T4>100% | N.a. | N.a. | No | N.a. | N.a. | N.a. | M |
| **Cys672Tyr** | III-17 |  | T4>100% | N.a. | N.a. | No | N.a. | N.a. | N.a. | M |
| **Cys672Tyr** | III-20 |  | T4>100% | N.a. | N.a. | No | N.a. | N.a. | N.a. | F |
| **Cys672Tyr** | III-21 |  | T4>100% | N.a. | N.a. | No | N.a. | N.a. | N.a. | M |
| **Cys672Tyr** | III-22 |  | T4>100% | N.a. | N.a. | No | N.a. | N.a. | N.a. | F |
| **Cys672Tyr** | III-25 |  | T4>100% | N.a. | N.a. | No | Tx | 19y | N.a. | F |
| **Cys672Tyr** | III-28 |  | T4>100% | N.a. | N.a. | No | N.a. | N.a. | N.a. | M |
| **Cys672Tyr** | IV-1 |  | T4>100% | N.a. | N.a. | No | MMI | 18 mo. | N.a. | F |
| **Cys672Tyr** | IV-4 |  | T4>100% | N.a. | N.a. | No | N.a. | N.a. | N.a. | F |
| **Ile691Phe** | II-4 | (26) | T4=131%  T3=243% | N.a. | N.a. | N.a. | N.a. | N.a. | N.a. | F |
| **Ile691Phe** | III-1 |  | T4=113%  T3=220% | N.a. | N.a. | N.a. | N.a. | N.a. | N.a. | F |
| **Ile691Phe** | III-4 |  | T4=369%  T3=193% | N.a. | N.a. | N.a. | N.a. | <5 | N.a. | M |
| **Ile691Phe** | III-7 |  | T4=263%  T3=145% | N.a. | N.a. | N.a. | N.a. | N.a. | N.a. | M |
| **Ile691Phe** | III-9 |  | T4=206%  T3=255% | N.a. | N.a. | N.a. | N.a. | N.a. | N.a. | M |
| **Ile691Phe** | III-12 |  | T4=450%  T3 normal | N.a. | N.a. | N.a. | N.a. | <5 | N.a. | F |
| **Ile691Phe** | IV-1 |  | T4=150%  T3=130% | N.a. | N.a. | N.a. | N.a. | N.a. | N.a. | F |
| **Ile691Phe** | IV-3 |  | T4=219%  T3=218% | N.a. | N.a. | N.a. | N.a. | 2 y | N.a. | F |
| **Ile691Phe** | IV-5 |  | T4=288%  T3=193% | N.a. | N.a. | N.a. | N.a. | <5 | N.a. | F |
| **Ile691Phe** | IV-9 |  | T4=213%  T3=180% | N.a. | N.a. | N.a. | N.a. | <5 | N.a. | M |

# References

1. Fukata S, Hishinuma A, Nakatake N, Tajiri J. A Japanese family with familial nonautoimmune hyperthyroidism with a novel mutation (Asn406Ser) in extracellular domain of thyrotrophin receptor. Clinical endocrinology. 2012;77(2):329-30.

2. Biebermann H, Schoneberg T, Hess C, Germak J, Gudermann T, Gruters A. The first activating TSH receptor mutation in transmembrane domain 1 identified in a family with nonautoimmune hyperthyroidism. The Journal of clinical endocrinology and metabolism. 2001;86(9):4429-33.

3. Elgadi A, Arvidsson CG, Janson A, Marcus C, Costagliola S, Norgren S. Autosomal-dominant non-autoimmune hyperthyroidism presenting with neuromuscular symptoms. Acta Paediatr. 2005;94(8):1145-8.

4. Winkler F, Kleinau G, Tarnow P, Rediger A, Grohmann L, Gaetjens I, et al. A new phenotype of nongoitrous and nonautoimmune hyperthyroidism caused by a heterozygous thyrotropin receptor mutation in transmembrane helix 6. The Journal of clinical endocrinology and metabolism. 2010;95(8):3605-10.

5. Supornsilchai V, Sahakitrungruang T, Wongjitrat N, Wacharasindhu S, Suphapeetiporn K, Shotelersuk V. Expanding clinical spectrum of non-autoimmune hyperthyroidism due to an activating germline mutation, p.M453T, in the thyrotropin receptor gene. Clinical endocrinology. 2009;70(4):623-8.

6. Fuhrer D, Warner J, Sequeira M, Paschke R, Gregory J, Ludgate M. Novel TSHR germline mutation (Met463Val) masquerading as Graves' disease in a large Welsh kindred with hyperthyroidism. Thyroid : official journal of the American Thyroid Association. 2000;10(12):1035-41.

7. Ferrara AM, Capalbo D, Rossi G, Capuano S, Del Prete G, Esposito V, et al. A new case of familial nonautoimmune hyperthyroidism caused by the M463V mutation in the TSH receptor with anticipation of the disease across generations: a possible role of iodine supplementation. Thyroid : official journal of the American Thyroid Association. 2007;17(7):677-80.

8. Akcurin S, Turkkahraman D, Tysoe C, Ellard S, De Leener A, Vassart G, et al. A family with a novel TSH receptor activating germline mutation (p.Ala485Val). European journal of pediatrics. 2008;167(11):1231-7.

9. Tonacchera M, Van Sande J, Cetani F, Swillens S, Schvartz C, Winiszewski P, et al. Functional characteristics of three new germline mutations of the thyrotropin receptor gene causing autosomal dominant toxic thyroid hyperplasia. The Journal of clinical endocrinology and metabolism. 1996;81(2):547-54.

10. Pohlenz J, Pfarr N, Kruger S, Hesse V. Subclinical hyperthyroidism due to a thyrotropin receptor (TSHR) gene mutation (S505R). Acta Paediatr. 2006;95(12):1685-7.

11. Vaidya B, Campbell V, Tripp JH, Spyer G, Hattersley AT, Ellard S. Premature birth and low birth weight associated with nonautoimmune hyperthyroidism due to an activating thyrotropin receptor gene mutation. Clinical endocrinology. 2004;60(6):711-8.

12. Duprez L, Parma J, Van Sande J, Allgeier A, Leclere J, Schvartz C, et al. Germline mutations in the thyrotropin receptor gene cause non-autoimmune autosomal dominant hyperthyroidism. Nature genetics. 1994;7(3):396-401.

13. Karges B, Krause G, Homoki J, Debatin KM, de Roux N, Karges W. TSH receptor mutation V509A causes familial hyperthyroidism by release of interhelical constraints between transmembrane helices TMH3 and TMH5. The Journal of endocrinology. 2005;186(2):377-85.

14. Claus M, Maier J, Paschke R, Kujat C, Stumvoll M, Fuhrer D. Novel thyrotropin receptor germline mutation (Ile568Val) in a Saxonian family with hereditary nonautoimmune hyperthyroidism. Thyroid : official journal of the American Thyroid Association. 2005;15(9):1089-94.

15. Nishihara E, Chen CR, Higashiyama T, Mizutori-Sasai Y, Ito M, Kubota S, et al. Subclinical nonautoimmune hyperthyroidism in a family segregates with a thyrotropin receptor mutation with weakly increased constitutive activity. Thyroid : official journal of the American Thyroid Association. 2010;20(11):1307-14.

16. Alberti L, Proverbio MC, Costagliola S, Weber G, Beck-Peccoz P, Chiumello G, et al. A novel germline mutation in the TSH receptor gene causes non-autoimmune autosomal dominant hyperthyroidism. European journal of endocrinology / European Federation of Endocrine Societies. 2001;145(3):249-54.

17. Nishihara E, Nagayama Y, Amino N, Hishinuma A, Takano T, Yoshida H, et al. A novel thyrotropin receptor germline mutation (Asp617Tyr) causing hereditary hyperthyroidism. Endocrine journal. 2007;54(6):927-34.

18. Schwab KO, Gerlich M, Broecker M, Sohlemann P, Derwahl M, Lohse MJ. Constitutively active germline mutation of the thyrotropin receptor gene as a cause of congenital hyperthyroidism. The Journal of pediatrics. 1997;131(6):899-904.

19. Ringkananont U, Van Durme J, Montanelli L, Ugrasbul F, Yu YM, Weiss RE, et al. Repulsive separation of the cytoplasmic ends of transmembrane helices 3 and 6 is linked to receptor activation in a novel thyrotropin receptor mutant (M626I). Mol Endocrinol. 2006;20(4):893-903.

20. Jaeschke H, Eszlinger M, Lueblinghoff J, Coslovsky R, Paschke R. Prolonged inappropriate TSH suppression during hypothyroidism after thyroid ablation in a patient with nonautoimmune familial hyperthyroidism. Hormone and metabolic research = Hormon- und Stoffwechselforschung = Hormones et metabolisme. 2011;43(7):500-4.

21. Fuhrer D, Wonerow P, Willgerodt H, Paschke R. Identification of a new thyrotropin receptor germline mutation (Leu629Phe) in a family with neonatal onset of autosomal dominant nonautoimmune hyperthyroidism. The Journal of clinical endocrinology and metabolism. 1997;82(12):4234-8.

22. Nwosu BU, Gourgiotis L, Gershengorn MC, Neumann S. A novel activating mutation in transmembrane helix 6 of the thyrotropin receptor as cause of hereditary nonautoimmune hyperthyroidism. Thyroid : official journal of the American Thyroid Association. 2006;16(5):505-12.

23. Biebermann H, Schoneberg T, Krude H, Gudermann T, Gruters A. Constitutively activating TSH-receptor mutations as a molecular cause of non-autoimmune hyperthyroidism in childhood. Langenbeck's archives of surgery / Deutsche Gesellschaft fur Chirurgie. 2000;385(6):390-2.

24. Khoo DH, Parma J, Rajasoorya C, Ho SC, Vassart G. A germline mutation of the thyrotropin receptor gene associated with thyrotoxicosis and mitral valve prolapse in a Chinese family. The Journal of clinical endocrinology and metabolism. 1999;84(4):1459-62.

25. Tonacchera M, Cetani F, Costagliola S, Van Sande J, Refetoff S, Vassart G. Functional characteristics of a variant thyrotropin receptor. European journal of biochemistry / FEBS. 1996;238(2):490-4.

26. Liu Z, Sun Y, Dong Q, He M, Cheng CH, Fan F. A novel TSHR gene mutation (Ile691Phe) in a Chinese family causing autosomal dominant non-autoimmune hyperthyroidism. Journal of human genetics. 2008;53(5):475-8.
