# Supplementary material for: A new family with an activating mutation (G431S) in the TSH receptor gene: a phenotype discussion and review of the literature
Source: Int J Pediatr Endocrinol. 2014 Nov 17;2014(1):23. doi: 10.1186/1687-9856-2014-23 (PMC4396564; doi:10.1186/1687-9856-2014-23)
Supplement: Supplementary file 2 — Additional file 2: Table S2: Summary of all reported patients with sporadic gain of function mutations in the TSHR. (DOCX 111 KB) [file 13633_2014_366_MOESM2_ESM.docx]

# Additional file 2: Table S2

| **Summary of all reported patients with sporadic gain of function mutations in the TSH Receptor** | | | | | | | | | | |
| --- | --- | --- | --- | --- | --- | --- | --- | --- | --- | --- |
| ***Mutation*** | ***Reference*** | ***Free T4 and free T3 (% upper limit)*** | ***Preterm birth (<37wks)*** | ***Weight at birth***  ***(Gram)*** | ***Developmental problems (IQ test, speech delay)*** | ***Prominent eyes*** | ***Treatment*** | ***Age of diagnosis*** | ***Craniosynostosis***  ***Head circumference (cm)*** | ***F/M*** |
| **Ser281Asn** | (1) | T4>350%  T3=192% | No | 2410 | N.a. | No | MMI, Tx (6.9) | Birth | Yes | F |
| **Ser281Asn** | (2) | T4=405%  T3=416% | Yes, 36 | 2520 | N.a. | No | MMI, Tx (6y) | 4 mo | Yes | F |
| **Ser281Asn** | (3) | T4>857%  T3>541% | Yes, 34 | 2350 | N.a. | Yes | Potassium Iodide, PTU, | 4 mo | Yes | M |
| **Ala428Val** | (4) | T4=209%  T3= 263% | No | 2550 | N.a. | N.a. | MMI | 26 d | 31.4 (5^th^) | F |
| **Met453Thr** | (5) | T4>418%  T3>546% | Yes, 32 | 1690 | N.a. | Yes | ATD | Birth | No | M |
| **Met453Thr** | (6) | TT4=326%  TT3=357% | No | 3040 | Yes but improved with treatment | Yes | ATD, Tx (7y), RAI (4 times between 9 to 13 y) | 7 mo | N.a. | M |
| **Ile486Asn** | (7) | T4>410%  TT3= 275% | Yes, 36 | 1 mo, 3085 | N.a. | N.a. | MMI | 1 Mo | N.a. | N.a. |
| **Ser505Asn** | (8) | T4=407%  T3=315% | No | 2600 | Yes (caught up MD but not SD) | Yes mild | MMI, PTU, Tx (25 mo) | 5 Mo | Yes | M |
| **Ser505Asn** | (9) | TT4= 345%  TT3=332% | No | 2540 | No | No | MMI | 11 mo | N.a. | F |
| **Leu512Gln** | (10) | TT4=361 %  TT3= 350% | Yes, 32 | 1860 | Yes | N.a. | MMI | 10 d | Yes | F |
| **Ile568Thr** | (11) | T4=1041%  T3=425% | Yes, 35 | 2050 | No | Yes | PTU | 38 d | No | F |
| **Ile568Thr** | (12) | N.a. | Yes, 35 | N.a. | N.a. | N.a. | PTU | 10d | N.a. | M |
| **Val597Leu** | (13) | T4=504%  T3=494% | No | 2500 | N.a. | N.a. | MMI, Tx (14. mo) | 9 mo | 43.2 (9^th^) | F |
| **Ile630Leu** | (14) | T4=270%  T3=248% | No | 2700 | No | Yes | PTU, Tx (11y), RAI (15y) | 10 mo | No | M |
| **Phe631Leu** | (15) | TT4=203%  TT3= 150% | Yes, 32 | 1600 | Yes | N.a. | PTU, MMI, Tx (8.7y), RAI | Birth | 29 | M |
| **Thr632Ile** | (16) | TT4=162% | Yes, 33 | 1450 | Yes | N.a. | PTU, Tx (3y) | Birth | Yes | F |
| **Asp633Tyr** | (17) | N.a. | Yes, 36 | 2400 | No | Yes | MMI, Tx (10y), Tx (13y), PTU, Tx (20y) | 6 mo | Yes | M |
| **Cys636Arg** | (18) | T4=103%  T3=219% | N.a. | N.a. | N.a. | N.a. | MMI | 12 y | N.a. | F |
| **Pro639Ser** | (19) | T4=167%  T3=339% | No | 4000g | No | N.a. | MMI | 24 mo | No | F |
| **Asn670Ser** | (20) | Normal values | N.a. | N.a. | N.a. | N.a. | RAI, MMI | 74 year | N.a. | F |
| **Abbreviations: MMI=Methimazole, PTU= propylthiouracil, Tx=thyroidectomy, RAI=Radioiodine treatment, N.a.=not available, TT4=total T4, M=Male, F=Female, Mo= Months, Y=years, Wks= weeks, ATD=antithyroid drugs, MD= motor delay, SD= speech delay** | | | | | | | | | | |

# References

1. Scaglia PA, Chiesa A, Bastida G, Pacin M, Domene HM, Gruneiro-Papendieck L. Severe congenital non-autoimmune hyperthyroidism associated to a mutation in the extracellular domain of thyrotropin receptor gene. Arquivos brasileiros de endocrinologia e metabologia. 2012;56(8):513-8.

2. Gruters A, Schoneberg T, Biebermann H, Krude H, Krohn HP, Dralle H, et al. Severe congenital hyperthyroidism caused by a germ-line neo mutation in the extracellular portion of the thyrotropin receptor. The Journal of clinical endocrinology and metabolism. 1998;83(5):1431-6.

3. Chester J, Rotenstein D, Ringkananont U, Steuer G, Carlin B, Stewart L, et al. Congenital neonatal hyperthyroidism caused by germline mutations in the TSH receptor gene. Journal of pediatric endocrinology & metabolism : JPEM. 2008;21(5):479-86.

4. Borgel K, Pohlenz J, Koch HG, Bramswig JH. Long-term carbimazole treatment of neonatal nonautoimmune hyperthyroidism due to a new activating TSH receptor gene mutation (Ala428Val). Hormone research. 2005;64(4):203-8.

5. de Roux N, Polak M, Couet J, Leger J, Czernichow P, Milgrom E, et al. A neomutation of the thyroid-stimulating hormone receptor in a severe neonatal hyperthyroidism. The Journal of clinical endocrinology and metabolism. 1996;81(6):2023-6.

6. Lavard L, Sehested A, Brock Jacobsen B, Muller J, Perrild H, Feldt-Rasmussen U, et al. Long-term follow-Up of an infant with thyrotoxicosis due to germline mutation of the TSH receptor gene (Met453Thr). Hormone research. 1999;51(1):43-6.

7. Biebermann H, Winkler F, Handke D, Gruters A, Krude H, Kleinau G. Molecular description of non-autoimmune hyperthyroidism at a neonate caused by a new thyrotropin receptor germline mutation. Thyroid research. 2011;4 Suppl 1:S8.

8. Holzapfel HP, Wonerow P, von Petrykowski W, Henschen M, Scherbaum WA, Paschke R. Sporadic congenital hyperthyroidism due to a spontaneous germline mutation in the thyrotropin receptor gene. The Journal of clinical endocrinology and metabolism. 1997;82(11):3879-84.

9. Fuhrer D, Mix M, Wonerow P, Richter I, Willgerodt H, Paschke R. Variable phenotype associated with Ser505Asn-activating thyrotropin-receptor germline mutation. Thyroid : official journal of the American Thyroid Association. 1999;9(8):757-61.

10. Nishihara E, Fukata S, Hishinuma A, Kudo T, Ohye H, Ito M, et al. Sporadic congenital hyperthyroidism due to a germline mutation in the thyrotropin receptor gene (Leu 512 Gln) in a Japanese patient. Endocrine journal. 2006;53(6):735-40.

11. Tonacchera M, Agretti P, Rosellini V, Ceccarini G, Perri A, Zampolli M, et al. Sporadic nonautoimmune congenital hyperthyroidism due to a strong activating mutation of the thyrotropin receptor gene. Thyroid : official journal of the American Thyroid Association. 2000;10(10):859-63.

12. Watkins MG, Dejkhamron P, Huo J, Vazquez DM, Menon RK. Persistent neonatal thyrotoxicosis in a neonate secondary to a rare thyroid-stimulating hormone receptor activating mutation: case report and literature review. Endocrine practice : official journal of the American College of Endocrinology and the American Association of Clinical Endocrinologists. 2008;14(4):479-83.

13. Esapa CT, Duprez L, Ludgate M, Mustafa MS, Kendall-Taylor P, Vassart G, et al. A novel thyrotropin receptor mutation in an infant with severe thyrotoxicosis. Thyroid : official journal of the American Thyroid Association. 1999;9(10):1005-10.

14. Bertalan R, Sallai A, Solyom J, Lotz G, Szabo I, Kovacs B, et al. Hyperthyroidism caused by a germline activating mutation of the thyrotropin receptor gene: difficulties in diagnosis and therapy. Thyroid : official journal of the American Thyroid Association. 2010;20(3):327-32.

15. Kopp P, van Sande J, Parma J, Duprez L, Gerber H, Joss E, et al. Brief report: congenital hyperthyroidism caused by a mutation in the thyrotropin-receptor gene. The New England journal of medicine. 1995;332(3):150-4.

16. Kopp P, Jameson JL, Roe TF. Congenital nonautoimmune hyperthyroidism in a nonidentical twin caused by a sporadic germline mutation in the thyrotropin receptor gene. Thyroid : official journal of the American Thyroid Association. 1997;7(5):765-70.

17. Bircan R, Miehle K, Mladenova G, Ivanova R, Ivanova R, Sarafova A, et al. Multiple relapses of hyperthyroidism after thyroid surgeries in a patient with long term follow-up of sporadic non-autoimmune hyperthyroidism. Experimental and clinical endocrinology & diabetes : official journal, German Society of Endocrinology [and] German Diabetes Association. 2008;116(6):341-6.

18. Biebermann H, Winkler F, Handke D, Teichmann A, Gerling B, Cameron F, et al. New pathogenic thyrotropin receptor mutations decipher differentiated activity switching at a conserved helix 6 motif of family A GPCR. The Journal of clinical endocrinology and metabolism. 2012;97(2):E228-32.

19. Agretti P, De Marco G, Biagioni M, Iannilli A, Marigliano M, Pinchera A, et al. Sporadic congenital nonautoimmune hyperthyroidism caused by P639S mutation in thyrotropin receptor gene. European journal of pediatrics. 2012;171(7):1133-7.

20. Schaarschmidt J, Paschke S, Ozerden M, Jaschke H, Huth S, Eszlinger M, et al. Late manifestation of subclinical hyperthyroidism after goitrogenesis in an index patient with a N670S TSH receptor germline mutation masquerading as TSH receptor antibody negative Graves' disease. Hormone and metabolic research = Hormon- und Stoffwechselforschung = Hormones et metabolisme. 2012;44(13):962-5.
